# Supplementary material for: Effect of Phenolics from Aeonium arboreum on Alpha Glucosidase, Pancreatic Lipase, and Oxidative Stress; a Bio-Guided Approach
Source: Pharmaceutics. 2023 Oct 27;15(11):2541. doi: 10.3390/pharmaceutics15112541 (PMC10675073; doi:10.3390/pharmaceutics15112541)
Supplement: Supplementary file 1 [file pharmaceutics-15-02541-s001.zip › pharmaceutics-2635165-supplementary.pdf]

Article

Effect of Phenolics from *Aeonium arboreum* on Alpha Glucosidase, Pancreatic Lipase, and Oxidative Stress; a Bio-Guided Approach

Marwah M. Alfeqy [1,*], Seham S. El-Hawary [2], Ali M. El-Halawany [2], Mohamed A. Rabeh [3], Saad A. Alshehri [3], Aya M. Serry [4], Heba A. Fahmy [1,†] and Marwa. I. Ezzat [2,*,†]

[1] Pharmacognosy Department, Faculty of Pharmacy, Modern University for Technology & Information, Cairo 11571, Egypt; heba.fahmy@pharm.mti.edu.eg

[2] Pharmacognosy Department, Faculty of Pharmacy, Cairo University, Kasr El Aini, Cairo 11562, Egypt; seham.elhawary@pharma.cu.edu.eg (S.S.E.-H.); ali.elhalawany@pharma.cu.edu.eg (A.M.E.-H.)

[3] Pharmacognosy Department, College of Pharmacy, King Khalid University, Abha 62251, Saudi Arabia; mrabeh@kku.edu.sa (M.A.R.); salshhri@kku.edu.sa (S.A.A.)

[4] Pharmaceutical Chemistry Department, Faculty of Pharmacy, Modern University for Technology & Information, Cairo 11571, Egypt; ayaserry@hotmail.com

* Correspondence: drmarwahalfeqy@gmail.com (M.M.A.); marwa.ezzat@pharma.cu.edu.eg (M.I.E.)

† These authors contributed equally to this work.

Supplementary data

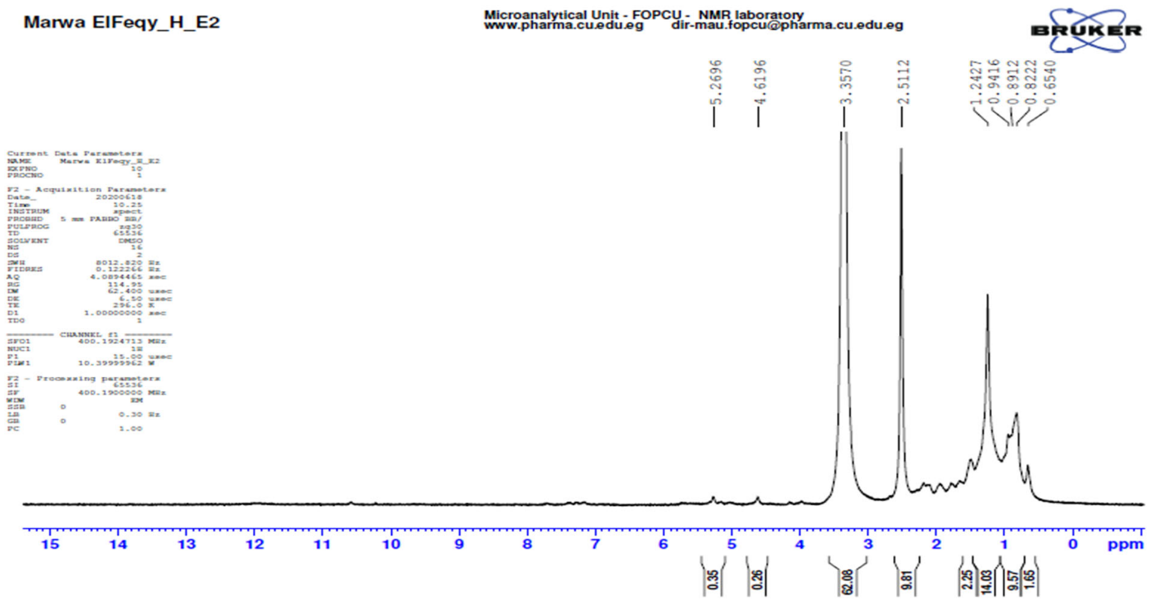

Figure S1. ^{1}H NMR Chart of β-sitosterol (DMSO, 400 MHz)**.**

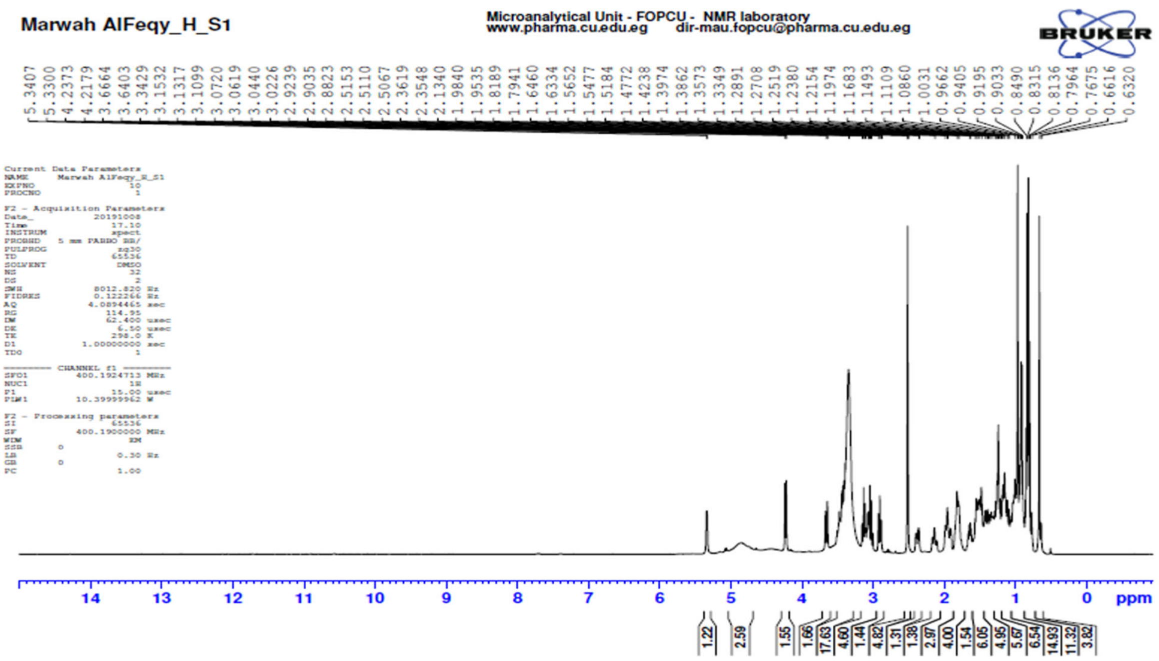

Figure S2. ^{1}H NMR Chart of β-sitosterol glucoside (DMSO, 400 MHz).

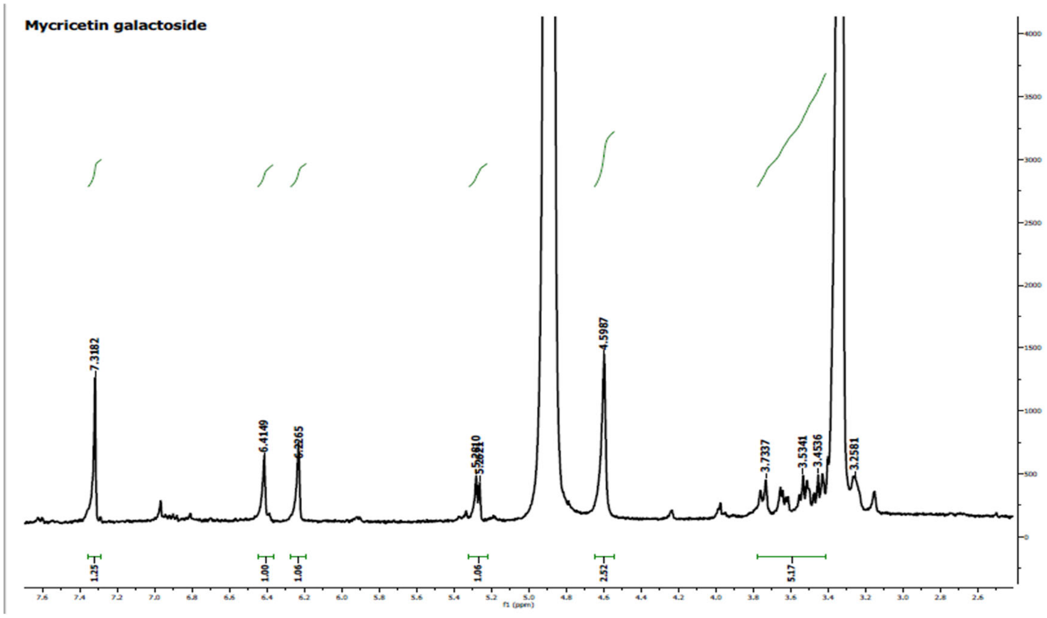

Figure S3. ^{1}H NMR Chart of myricetin galactoside (MeOD, 400 MHz).

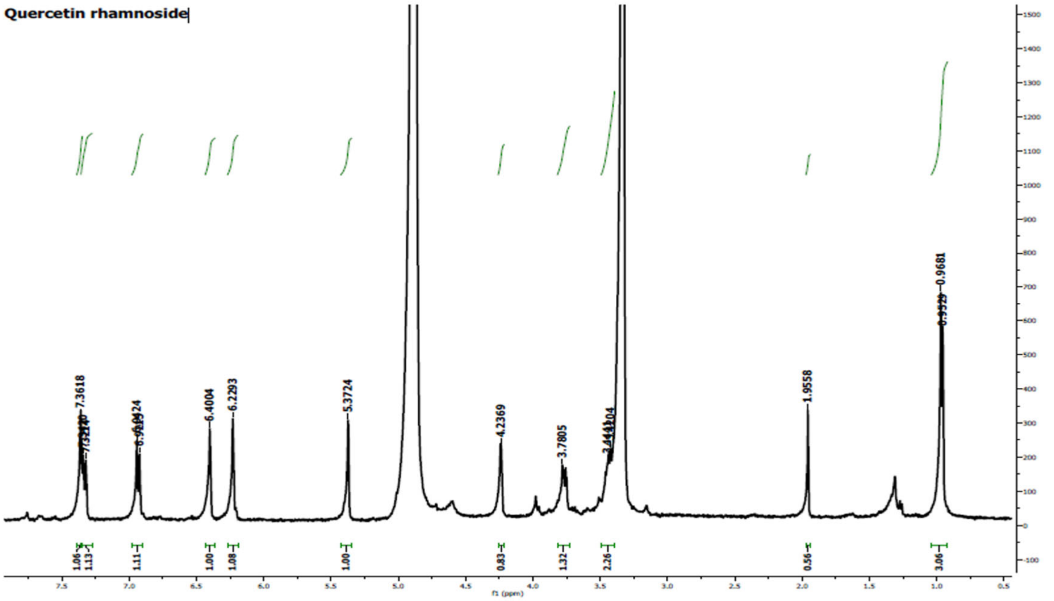

Figure S4. ^{1}H NMR Chart of quercetin rhamnoside (MeOD, 400 MHz).

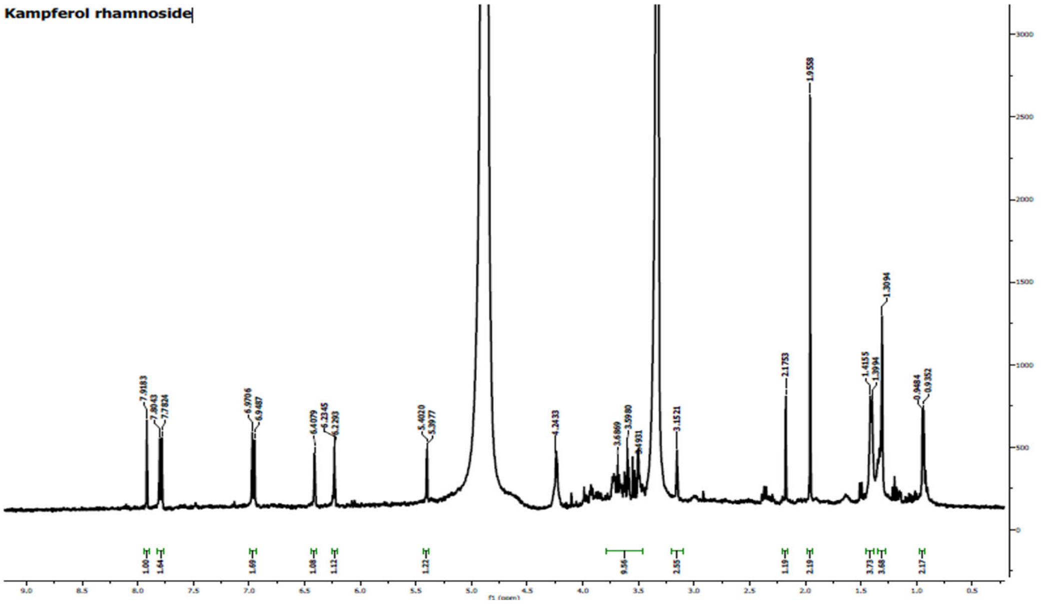

Figure S5. ^{1}H NMR Chart of kampferol rhamnoside (MeOD, 400 MHz).

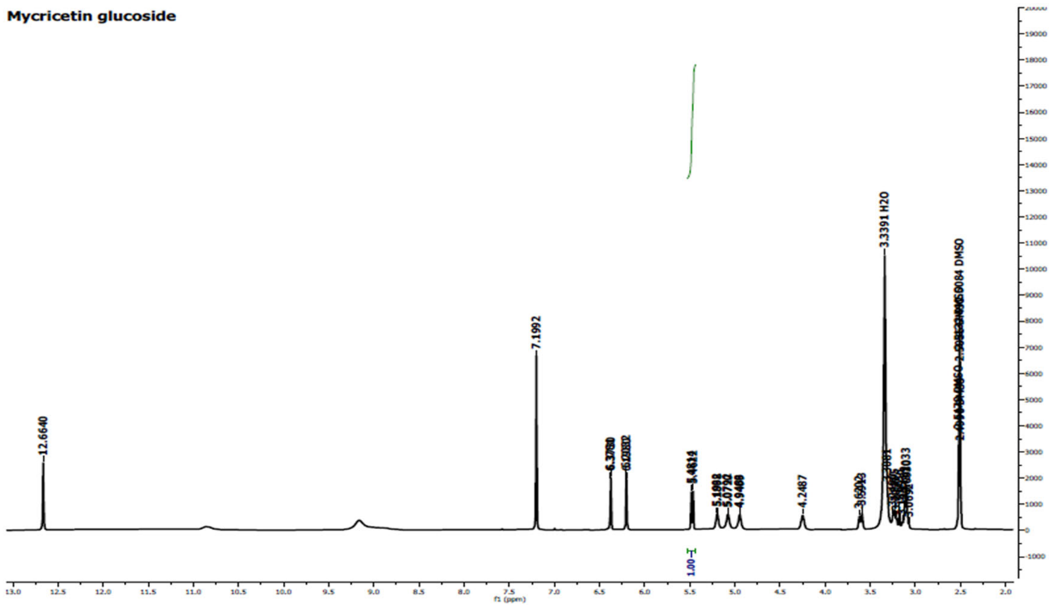

Figure S6. ^{1}H NMR Chart of myricetin glucoside (DMSO, 400 MHz).

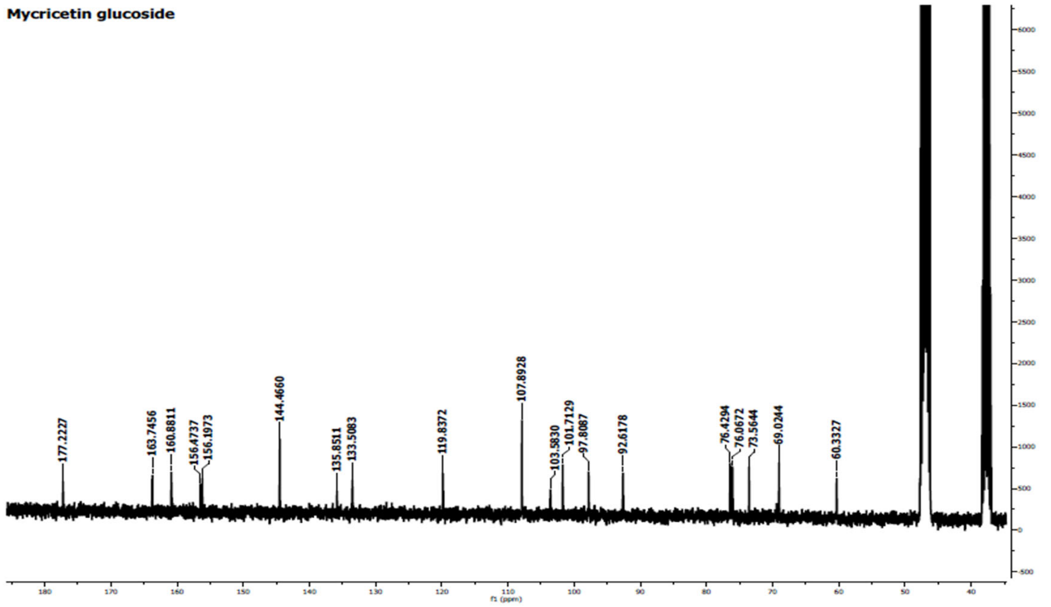

Figure S7. ^{13}C NMR Chart of myricetin glucoside (DMSO, 400 MHz).

Table S1. α-glucosidase inhibitory effect of *A. arboreum* fractions and acarbose.

Sample	Final concentrations used (μg/mL)	IC_{50} (Mean ± SE)
MeOH extract	-	> 1000 μg/mL
DCM fraction	100, 150, 200, 300 and 400	147.90 ± 1.03 μg/mL
100 % MeOH diaion fraction	12.5, 25, 50, 100 and 200	48.24 ± 1.02μg/mL
50 % MeOH diaion fraction	12.5, 25, 50, 100 and 200	44.26 ± 1.06 μg/mL
(Quercetin-3-rhamnoside)	25, 50, 100, 200 and 400	126.40 ± 1.04 μM
Acarbose	31.25, 62.5, 125, 250, 500, 750 and 1000	161.40 ± 1.05 μM

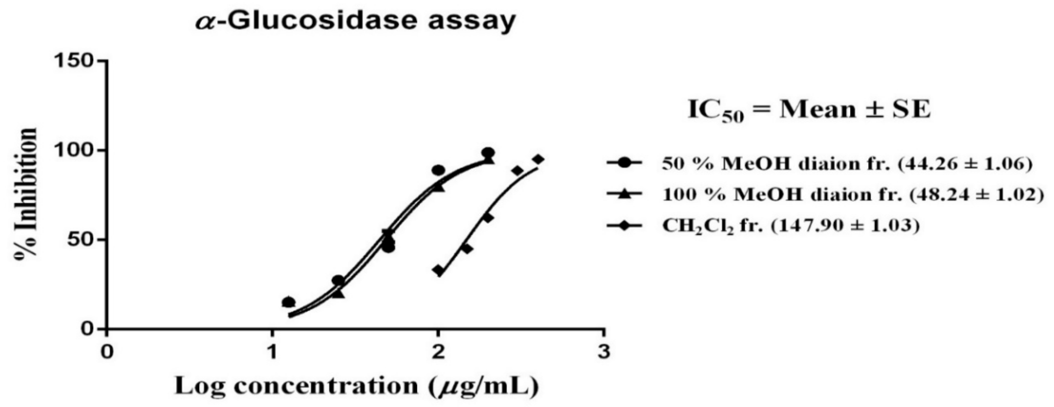

Figure S8. a) Inhibitory effect of A. arboreum fractions on α-glucosidase**.**

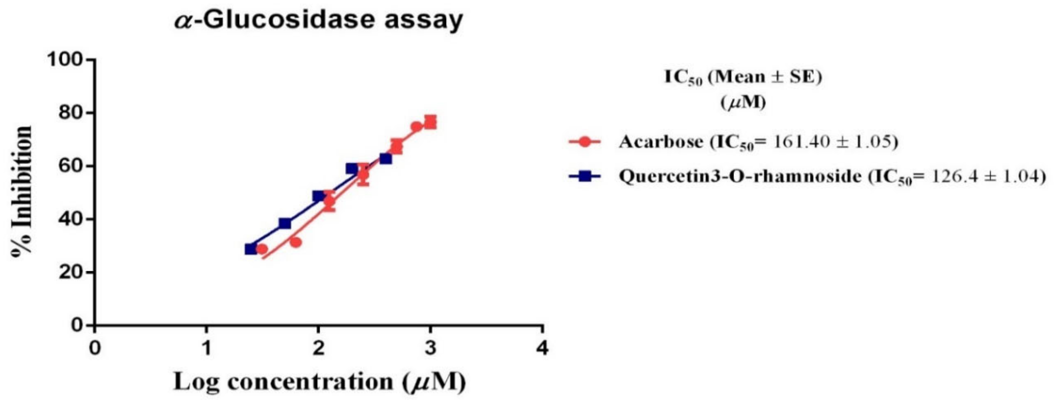

Figure S8. b) Inhibitory effect of quercetin rhamnoside isolated from A. arboreum on α-glucosidase in comparison with acarbose.

Table S2. Pancreatic lipase inhibitory effect of *A. arboreum* fractions and orlistat.

Sample	% inhibition (Mean ± SD) (500 μg/mL or μM)	IC_{50} (μM) (Mean ± SE)
MeOH extract	NO	More than 500 μg/mL
DCM fraction	23.05 ± 1.05	More than 500 μg/mL
100 % MeOH diaion fraction	9.43 ± 0.32	More than 500 μg/mL
50 % MeOH diaion fraction	4.88 ± 0.55	More than 500 μg/mL
(Quercetin-3-rhamnoside)	19.06 ± 0.34	More than 500 μM
Orlistat		0.70 ± 1.10 μM

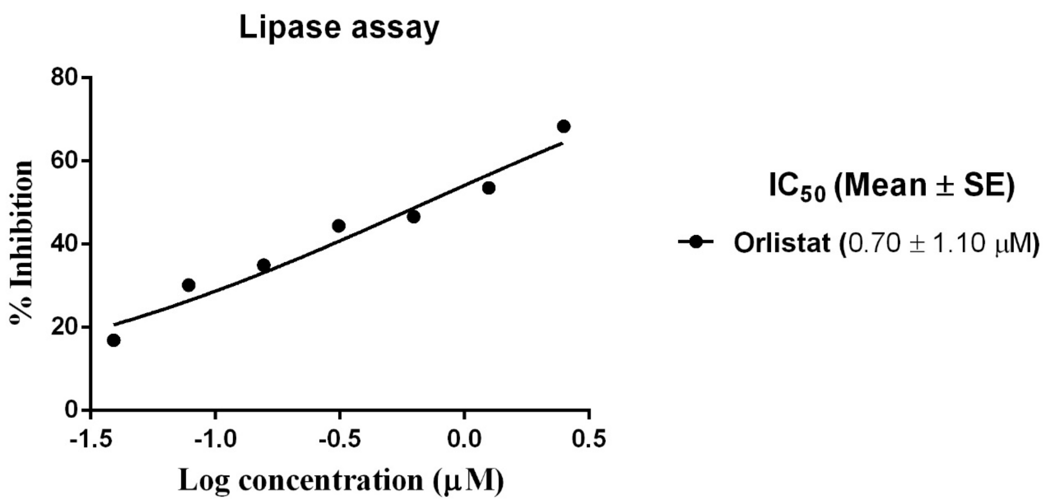

Figure S9. Pancreatic lipase inhibitory effect of orlistat.

Table S3. antioxidant activity of *A. arboreum* fractions using DPPH assay.

Sample I.D	Micro molar Trolox equivalent per mg extract (µM AAE/mg extract)	Standard deviation
MeOH extract	66.13	3.45
DCM fraction	313.23	10.53
100 % MeOH diaion fraction	824.06	65.68
50 % MeOH diaion fraction	984.71	93.28
(Quercetin-3-rhamnoside)	576.88	33.43

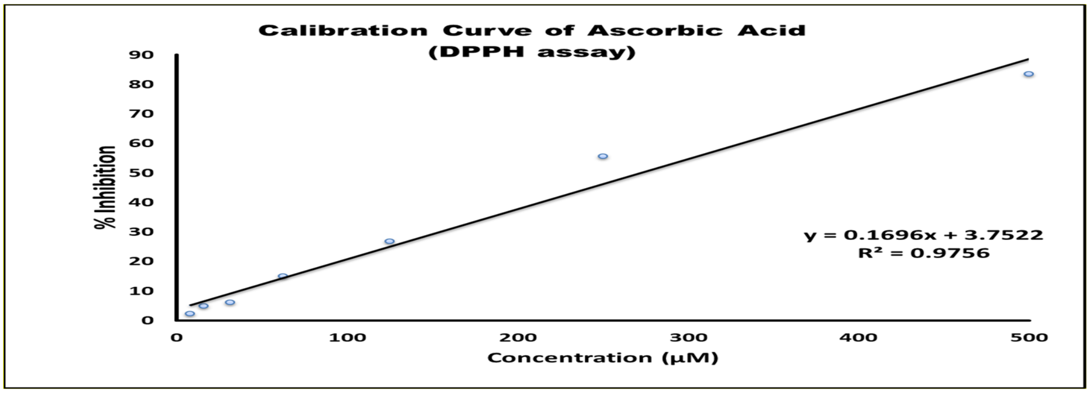

Figure S10. Concentration-response linear curve of ascorbic acid in DPPH assay.

Table S4. antioxidant activity of *A. arboreum* fractions using ABTS assay.

Sample I.D	Micro molar ascorbic acid equivalent per mg extract (µM AAE/mg extract)	Standard deviation
MeOH extract	141.1490	8.8030
DCM fraction	161.8987	6.0702
100 % MeOH diaion fraction	1126.8284	87.4056
50 % MeOH diaion fraction	862.3991	104.0465
(Quercetin-3-rhamnoside)	1338.0894	81.3854

Concentration (µM)	% inhibition
7.8125	2.8930
15.625	3.7803
31.25	5.5156
62.5	9.7291
125	16.88018
250	33.5451
500	62.85834

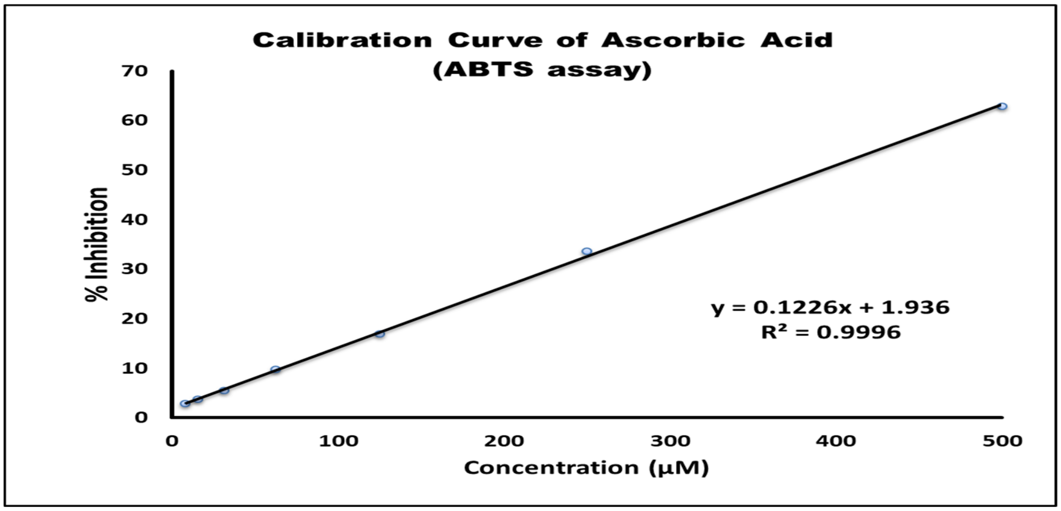

Figure S11. Concentration-response linear curve of ascorbic acid in ABTS assay.

Table S5. antioxidant activity of *A. arboreum* fractions using FRAP assay.

Sample I.D	Micro mole Trolox equivalent per mg sample (µM TE/mg sample)	Standard deviation
**MeOH extract**	**233.07**	**11.91**
**DCM fraction**	**237.18**	**20.19**
**100 % MeOH diaion fraction**	**669.08**	**33.63**
**50 % MeOH diaion fraction**	**574.75**	**51.28**
**(Quercetin-3-rhamnoside)**	**1681.23 µM TE/mM sample**	**49.91**

Concentration (µM)	Absorbance
200	0.350
300	0.569
400	0.709
500	0.829
600	0.966
700	1.149
800	1.2687
900	1.432
1200	1.812
1500	2.240

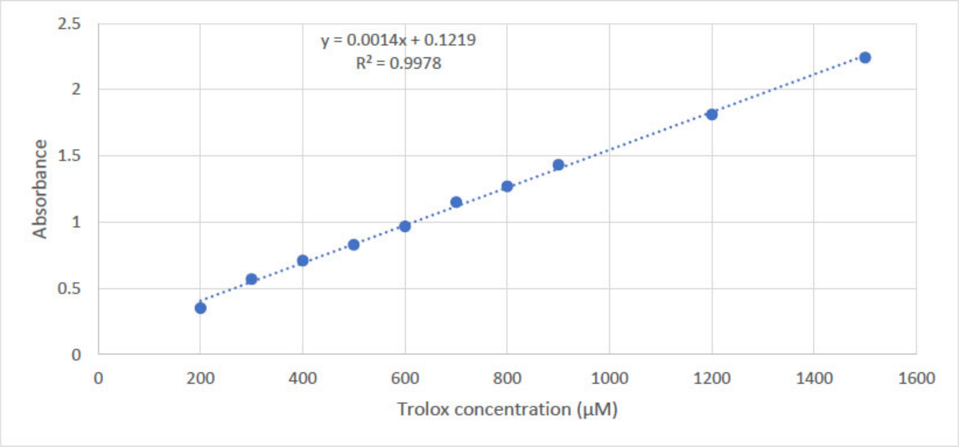

Figure S12. Concentration- response linear curve for solutions of Trolox in FRAP assay.

Table S6. antioxidant activity of *A. arboreum* fractions using ORAC assay.

Sample I.D	Antioxidant activity using ORAC assay is (μM TE /mg sample)	Standard deviation
**MeOH extract**	**9435.29**	**42.78**
**DCM fraction**	**16373.49**	**312.57**
**100 % MeOH diaion fraction**	**22140.5**	**1533.05**
**50 % MeOH diaion fraction**	**46781.3**	**3169.99**
**(Quercetin-3-rhamnoside)**	**25258.39 (μMT eq/mM)**	**1534.49**

Concentration (μM)	RFU
50	95512
100	154996
200	210115
300	291670
400	334351.5
500	414683
600	449383.3
700	496138
800	586368.3
1000	738391

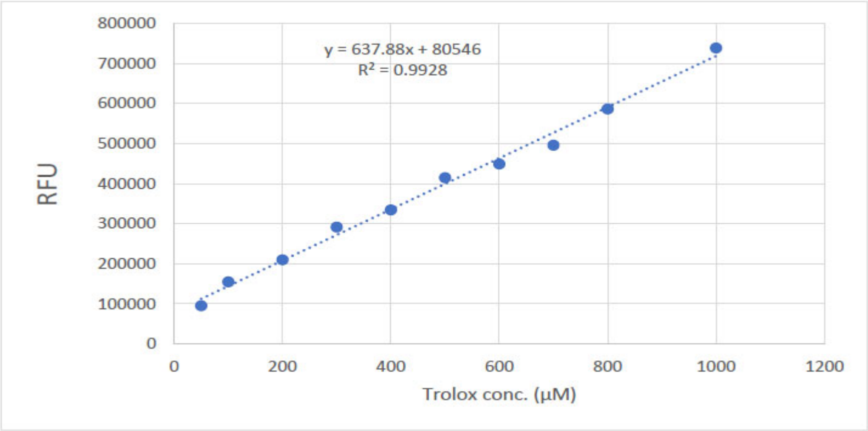

Figure (S13) Concentration -response linear curve of trolox in ORAC assay.

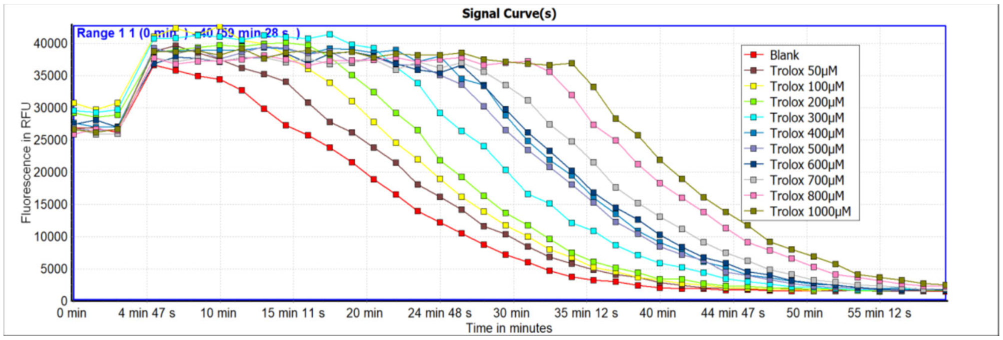

Figure S14. Antioxidant effect of Trolox on the decay of fluorescein in ORAC assay. (A) Blank corrected linear regression curve of Trolox. (B) Signal curves of different Trolox concentrations and blank indicating the decay of fluorescein with different concentrations of Trolox.

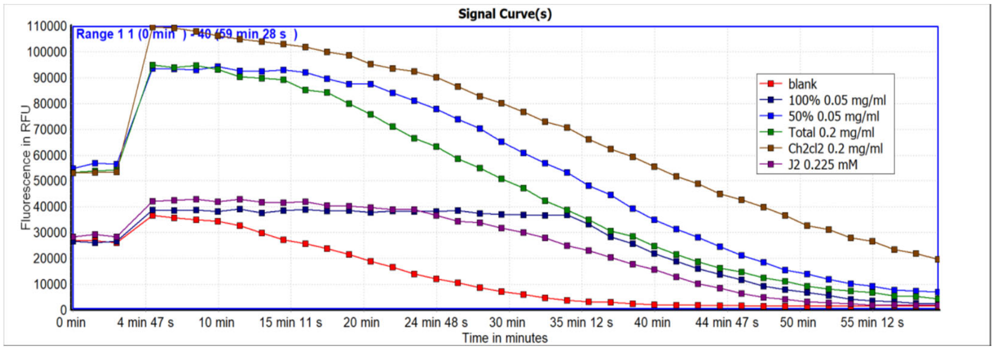

Figure S15. Signal curve of Samples and blank indicating the decay of fluorescein upon applying the samples.

Table S7. antioxidant activity of *A. arboreum* fractions using metal chelation assay.

Sample I.D	Micro molar EDTA equivalent per mg sample (µM EDTA eq/ mg sample	Standard deviation
MeOH extract	149.54	11.27
DCM fraction	361.94	29.27
100 % MeOH diaion fraction	331.12	10.17
50 % MeOH diaion fraction	291.14	8.52
(Quercetin-3-rhamnoside)	377.98 µM eq/ mM sample	26.97

Concentration (µM)	% inhibition
10	7.06
20	18.87
30	27.30
40	38.32
50	46.68
60	57.01
70	62.71
80	71.93

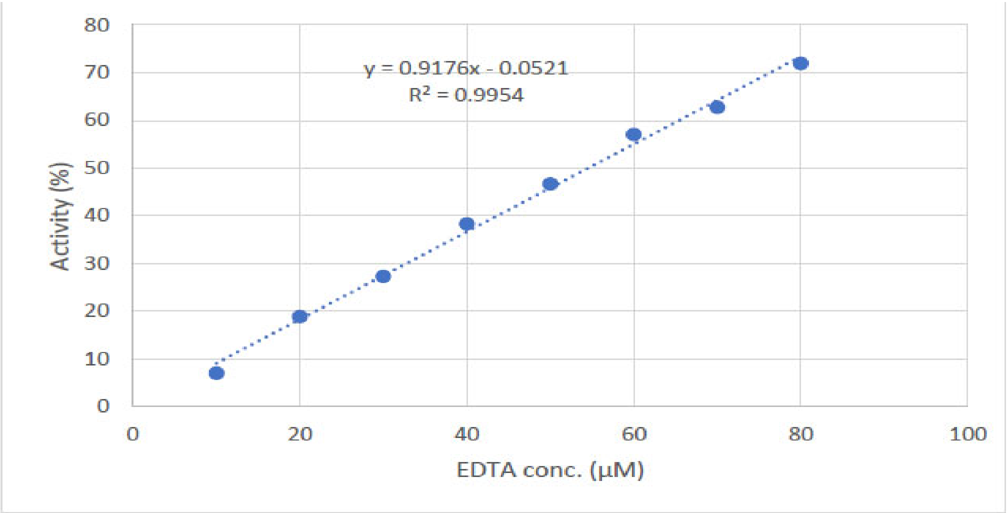

Figure S16. Concentration-response linear curve of EDTA in metal chelation assay.
